# Low-cost gelatin/collagen scaffolds for bacterial growth in bioreactors for biotechnology

**DOI:** 10.1007/s00253-025-13491-5

**Published:** 2025-05-08

**Authors:** Daniella Alejandra Pompa-Monroy, Ricardo Vera-Graziano, Syed G. Dastager, Graciela Lizeth Pérez-González, Nina Bogdanchikova, Ana Leticia Iglesias, Luis Jesús Villarreal-Gómez

**Affiliations:** 1 Facultad de Ciencias Químicas e Ingeniería, Universidad Autónoma de Baja California, Tijuana, Baja California, México; 2 Instituto de Investigaciones en Materiales, Universidad Nacional Autónoma de México, CDMX, México; 3 National Collection of Industrial Microorganisms (NCIM), CSIR-National Chemical Laboratory, Pune, Maharashtra India; 4 Centro de Nanociencias y Nanotecnología, Universidad Nacional Autónoma de México, Ensenada, Baja California, México; 5 Facultad de Ciencias de la Ingeniería y Tecnología, Universidad Autónoma de Baja California, Tijuana, Baja California, México

**Keywords:** Bacterial cell proliferation, Collagen, Gelatin, Polymeric electrospinning, Scaffolds

## Abstract

**Abstract:**

A wide array of pharmaceutical and industrial products available in today’s market stems from bioreactors. Meeting the escalating demand for these products necessitates significant enhancements in biotechnological processes. This study focuses on developing cost-effective scaffolds designed explicitly for use within bioreactors, employing commonly used polymers such as gelatin and collagen. Bacterial proliferation assays involving *Escherichia coli*, *Staphylococcus aureus*, and *Pseudomonas aeruginosa* were conducted to assess the effectiveness of these scaffolds. The scaffolds were produced by electrospinning polymeric solutions with varying concentrations of gelatin and collagen and were characterized using scanning electron microscopy, Fourier transform infrared spectroscopy, differential scanning calorimetry, and thermogravimetric analysis. Results revealed that scaffolds with 15% gelatin increased the 24-h proliferation of *S. aureus*, *P. aeruginosa*, and *E. coli* by 52%, 35%, and 20%, respectively. In the case of *E. coli*, scaffolds with lower gelatin concentrations (1–10%) were more effective, leading to 35–55% proliferation growth. These findings highlight the potential application of gelatin/collagen scaffolds in fabricating industrial products derived from these bacteria.

**Key points:**

*• GEL/COL fibers boost S. aureus growth by 128%*

*• Offers scalable biotech applications*

**Graphical Abstract:**

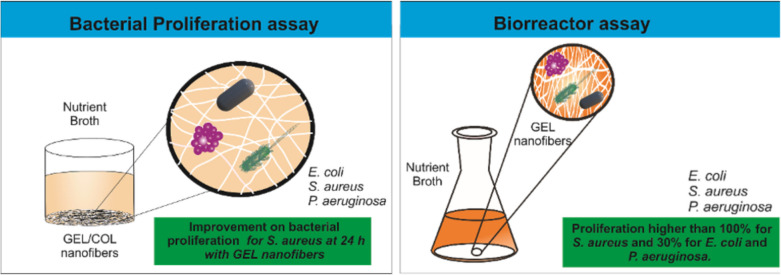

## Introduction

Microbial communities have emerged as an intriguing strategy for generating industrial, agricultural, and biomedical products. The evolutionary pressures faced by microbes have resulted in a vast array of species endowed with diverse metabolic and genetic potentials. However, despite this abundance, millions of microorganisms pose challenges in cultivating and harnessing their full potential (Sivasubramaniam and Franks 2016; Gonzalez and Aranda 2023).

Nanofiber-based scaffolds have gained increasing attention in biotechnology due to their ability to influence bacterial growth dynamics. Previous studies, such as Moffa et al. (2017), demonstrated that polystyrene nanofibers can support the proliferation of *Streptomyces lividans*, indicating that nanofibrous structures can provide a favorable microenvironment for bacterial colonization (Moffa et al. 2017). Additionally, research by Velasco-Barraza et al. (2018) highlighted the role of electrospun nanofibers in promoting bacterial biofilm formation, further suggesting their potential in industrial applications (Velasco-Barraza et al. 2018). Despite these findings, the question of how nanofiber composition and structural properties influence the growth of specific bacterial species is still emerging area in microbiology and requires careful study.

One proposed strategy to optimize the growth of intriguing microorganisms involves the utilization of three-dimensional scaffolds crafted from nano- and microfibers created through the electrospinning technique. These scaffolds serve as substrates in microbial cultures (Velasco-Barraza et al. 2018).

Electrospinning, a technique employed to produce nanoscale fibers, has garnered significant attention in biomedical fields (Venugopal et al. 2005; Kahdim et al. 2023); this process involves injecting a well-blended polymeric solution into a polar solvent while applying high voltage. This action leads to the creation of Taylor’s cone, allowing the formation of fibers on a collector positioned at the negative terminal of the electrical field (Villarreal-Gómez et al. 2014, 2016; Cornejo-Bravo et al. 2017; Torres-Martinez et al. 2018; Villarreal-Gómez and Pérez-González 2023), yet, there is limited understanding regarding how nanofiber scaffolds impact the growth of industrially relevant bacteria.

This research focuses on two natural polymers, collagen (COL) and gelatin (GEL), representing distinct variations of the same macromolecule. Gelatin (GEL) is a flexible, soluble protein derived from the partial hydrolysis of collagen (COL) (Okutan et al. 2014). These molecules find diverse applications in fields such as food, cosmetics, photography, medicine, microbiology, and cell cultures (Fischer et al. 2012; Zhao et al. 2015; Bhagwat and Dandge 2018). However, collagen (COL) has been effectively electrospun solely in combination with other polymers like hyaluronic acid; it has not been electrospun alone (Fischer et al. 2012), hydroxyethyl cellulose, poly (vinyl alcohol) (Blosi et al. 2021; Zulkifli et al. 2023), chitosan (Nokoorani et al. 2021), and poly (lactic-co-glycolic acid) (Kwak et al. 2016), among others, using fluorinated solvents. Additionally, it might be feasible to electrospun collagen (COL) fibers devoid of organic solvents by employing telopeptide-free collagen in a basic environment (Foltran et al. 2008; Jha et al. 2011; Lu and Guo 2018; Feng et al. 2022). Likewise, electrospinning unmodified collagen (COL) without alterations or employing fluorinated solvents has not been documented. Conversely, gelatin (GEL) nanofibers find applications as skin dressings or scaffolds for tissue engineering (Ersanli et al. 2023). An advantage of using this protein is the absence of a requirement for fluorinated solvents. Gelatin has been electrospun multiple times, either independently or in combination with other polymers, yielding positive outcomes (Erencia et al. 2015; Safdari et al. 2016; İnanç Horuz and Belibağlı 2017; Ehrmann 2021; Samyn et al. 2023; Dechojarassri et al. 2023).

Gelatin and collagen nanofibers, known for their biocompatibility and structural resemblance to the native extracellular matrix, possess properties that impact bacterial growth (Chiu et al. 2007; Chen et al. 2010). Their high surface area-to-volume ratios and porous structures facilitate bacterial adhesion and colonization, while their tunable mechanical properties and degradation rates influence bacterial access to nutrients and growth factors (Meng et al. 2007). The incorporation of bioactive agents, surface modifications for antimicrobial properties, and electrostatic interactions with bacterial cell surfaces further modulate bacterial interactions. Hydrophilicity enhances water retention, creating a conducive environment for bacterial proliferation, while cross-linking density affects scaffold stability and bacterial infiltration (Campiglio et al. 2020). Overall, careful design and engineering of these nanofibrous scaffolds are crucial for achieving industrial objectives while minimizing the risk of bacterial infections (El-Seedi et al. 2023).

Previous reports have highlighted the utilization of nanofibers as antimicrobial agents. Yet, there remains a limited understanding regarding their capacity to stimulate the growth of bacteria relevant to biotechnology. Moffa et al. (2017) employed polystyrene nanofibers dissolved in *N, N*-dimethylformamide to foster the growth of *Streptomyces lividans*, yielding promising outcomes, suggesting that nanofibers can serve as substrates for bacterial proliferation, thereby expanding their potential applications beyond antimicrobial agents (Moffa et al. 2017). The findings underscore the importance of exploring the interactions between nanofibers and bacteria in various fields, including biotechnology. Further research in this area could elucidate the underlying mechanisms by which nanofibers support bacterial growth and inform the development of novel materials for biotechnological applications (Pan et al. 2015).

The objective of this study is to create nanofibers using collagen (COL) and gelatin (GEL), which can potentially be used in bioreactors to produce various bioproducts that are important to biotechnology. The primary aim is to optimize the growth conditions of three industrial bacteria, namely, *Staphylococcus aureus*, *Escherichia coli*, and *Pseudomonas aeruginosa*, using electrospun COL/GEL nanofibers.

*Staphylococcus aureus*, a Gram-positive bacterium, is ubiquitously found in the environment, necessitating an enzyme called lysostaphin that can be anchored to collagen molecular structure for its proliferation, as observed in studies by (Ghasemian et al. 2015) and (Shan et al. 2019). On the other hand, *Escherichia coli,* a Gram-negative bacterium, boasts an extensive range of bioproducts in its regular pathways, such as acetate, ethanol, formate, and succinate, as evidenced in the research by Li et al. (2017).

Furthermore, *Pseudomonas aeruginosa*, known for producing siderophores—element-specific ligands that induce pH variation in solutions and enhance thorium and uranium chelation, according to findings by (Desouky et al. 2016)—also synthesizes various pigments. Among these, pyocyanin, a green–blue soluble pigment, serves as a biosensor in diverse fields like agriculture, medicine, and the environment, as highlighted in the research by Zhou et al. (2022).

This research introduces novelty on multiple fronts: firstly, it proposes the utilization of electrospun GEL/COL nanofibers as substrates in bioreactors to foster bacterial growth, presenting a fresh approach distinct from conventional methods (Massaglia et al. 2021). Secondly, it focuses on collagen (COL) and gelatin (GEL), natural polymers with diverse applications, particularly in biotechnology, where their electrospinning, either alone or combined, has been minimally explored (Sell et al. 2010). Thirdly, the study identifies a gap in the literature regarding the promotion of bacterial growth through nanofibers, contrasting with existing research that primarily explores inhibitory effects on cell growth. By addressing this gap, the research aims to provide valuable insights into optimizing bacterial growth with nanofiber scaffolds (Berdimurodov et al. 2023). Lastly, by focusing on three important industrial bacteria, the study aligns with the practical requirements of biotechnological applications. Understanding how GEL/COL nanofibers can enhance the growth of these bacteria holds significant potential for improving bioproduction processes, potentially reducing growth time and enhancing overall efficiency (Fu et al. 2014; Zhong 2020).

*Staphylococcus aureus*, *Escherichia coli*, and *Pseudomonas aeruginosa* are considered industrially relevant bacteria due to their diverse applications in biotechnology, pharmaceuticals, and environmental sciences. *E. coli* is widely used in recombinant protein production, enzyme synthesis, and biofuel generation, making it a cornerstone of industrial microbiology. *S. aureus* plays a significant role in bioprocessing, particularly in the production of medically relevant enzymes and antimicrobial compounds. *P. aeruginosa* is known for its ability to produce biosurfactants and biopolymers, which are valuable in bioremediation, agriculture, and biopharmaceutical applications. Understanding how these bacteria interact with nanofiber scaffolds is crucial for optimizing their growth in industrial bioreactors and enhancing their biotechnological applications (Piatek et al. 2023).

Gelatin is more economically convenient for large-scale applications due to its lower cost, wide availability, and established production processes. Fish collagen, while more expensive, is justified in specialized or high-value applications where its superior biocompatibility and other specific properties are required. Both materials are sustainable as they are derived from by-products, but the cost-effectiveness of using them will depend heavily on the application and market requirements. For many biotechnological purposes, gelatin may be the more affordable choice, while fish collagen is reserved for premium applications (Lv et al. 2019).

This study presents a unique approach that utilizes GEL/COL nanofibers as a microbial bioreactor to improve the growth of three important industrial bacteria. Our research has identified a gap in the existing studies focused on promoting bacterial cell growth using nanofibers, except for previous investigations conducted within our research team (Velasco-Barraza et al. 2018; Pompa-Monroy et al. 2020, 2022). On the other hand, many studies have reported inhibitory effects on cell growth (Maliszewska and Czapka 2022; Nachev et al. 2022; Abadi et al. 2022). The objective of the study is to examine the utilization of GEL/COL nanofibers as an innovative approach to amplify the growth of three industrially relevant bacteria (*Staphylococcus aureus, Escherichia coli*, and *Pseudomonas aeruginosa*) within microbial bioreactors. This work seeks to confront the challenge of efficiently tapping into the full potential of microbial communities for biotechnological advancements.

## Methods

### Materials

Extra-pure gelatin (VWR, HiMedia Laboratories, MW 50,000 Daltons), type I fish collagen (Aribun, MW 1000–3000 Daltons), and glacial acetic acid (Jalmek, ≥ 99% purity) were utilized in the electrospinning process without undergoing any prior modifications. All remaining chemicals are sourced from Sigma-Aldrich unless stated otherwise.

### Preparation of polymeric blends

To prepare for the electrospinning process, a solution of GEL at 15% w/v in acetic acid was created in borosilicate vials and stirred for 2 h at 50 °C (GEL). Additional solutions were then prepared by adding 1%, 5%, and 10% of COL (resulting in GEL/COL1, GEL/COL5, and GEL/COL10). The electrospinning of each sample took place within 14 days of its preparation.

### Electrospinning

The electrospinning setup for nanofiber production comprised a random collector and a plastic syringe with a 21 G × 40 mm needle, used to inject a 1 mL sample solution. The collector and injection needle were positioned 15 cm apart. The parameters for the procedure included 20 kV voltage, a flow rate of 0.80 mL/h, a temperature range of 20–32 °C, and a humidity level of 20–40%. Continuous monitoring of droplet formation at the needle tip was maintained throughout the procedure, and the electrospinning process was performed for 1.25 h.

### Scanning electron microscopy (SEM)

The morphology and diameter of the polymeric fibers were examined using an SEM (JEOL JSM 7600f field emission microscope). Given the non-conductive nature of the samples, preliminary preparation involving a thin gold layer was necessary for analysis; samples were sputter-coated with a thin layer of gold before imaging. The gold coating was applied using a sputter coater (model XYZ) at 10 mA for 90 s, resulting in a coating thickness of approximately 10 nm. This conductive layer minimized charging effects and enhanced imaging contrast. Images were captured using an accelerating voltage of 20 kV. Image J software was used to determine the nanofiber diameter, taking 30 measurements from at least two distinct images of each sample. Furthermore, the threshold function of the software was employed to calculate the samples’ porosity. Subsequently, the collected data were plotted using Minitab17.

### Fourier transform infrared spectroscopy (FTIR)

Attenuated total reflectance (ATR) Fourier transform infrared (FTIR) spectroscopy was performed using a diamond ATR crystal. The diamond crystal was selected for its durability, high refractive index, and ability to provide strong signal intensities across a broad spectral range. Sample spectra were collected in the range of 4000–400 cm⁻^1^ with a resolution of 4 cm⁻^1^, ensuring accurate identification of the functional groups present in the electrospun scaffolds.

### Thermogravimetric analysis (TGA)

The thermogravimetric analysis aimed to track the loss of polymer mass in terms of temperature variations. This examination was conducted using a TGA Q5500 (TA Instruments) apparatus, heating at 10 °C per min from room temperature to 700 °C within a nitrogen atmosphere, utilizing an unsealed platinum tray. The resulting thermogram was then analyzed via the TA universal analysis software.

### Differential scanning calorimetry (DSC)

Differential scanning calorimetry (DSC) was utilized to determine both the glass transition temperature (Tm) and the degradation temperature (Td). This assessment was conducted using the TA Instruments DSC Q5000, employing unsealed aluminum trays with a heating rate of 10 °C per min, heating from room temperature to 300 °C under a nitrogen atmosphere. The resulting thermogram was analyzed using the TA universal analysis software.

### Culture medium preparation, inoculation, and adjustment

Bacterial strains such as *Escherichia coli* (ATCC 25922), *Pseudomonas aeruginosa* (ATCC 27853), and *Staphylococcus aureus* (ATCC 25923) were cultivated in nutrient broth no. 1 (Sigma-Aldrich) and minimal salt broth (MSB) (Sigma-Aldrich). Nutrient broth no. 1 (Oxoid, CM0001) was prepared according to the manufacturer’s instructions, consisting of peptone (10 g/L), beef extract (10 g/L), and sodium chloride (5 g/L), dissolved in distilled water and autoclaved at 121 °C for 15 min. Modified starch-based (MSB) medium was prepared following standard formulations, containing soluble starch (10 g/L), tryptone (5 g/L), yeast extract (5 g/L), dipotassium phosphate (2 g/L), magnesium sulfate (0.2 g/L), and sodium chloride (5 g/L), adjusted to pH 7.2 before sterilization. The broths were dissolved in distilled water in 25-mL Erlenmeyer flasks (one for each bacteria strain) and sterilized at 121 °C for 15 min. The cryopreserved strains (0.5 mL) were cultivated in each prepared media and incubated for 18 h at 35 °C. The obtained bacterial suspensions were used to subsequently take aliquots that were adjusted in nephelometer tubes to 0.5 McFarland with sterile saline water.

### Bacterial proliferation assay

To test the effect of the fibers on bacterial cultures, 5-mm diameter circles of the fibers were placed in triplicate on a sterile 96-well plate; 150 µL of sterile nutrient broth and 50 µL of cell suspension of each bacteria strain adjusted to 0.5 of the McFarland nephelometer were added. The McFarland standard was prepared and verified to ensure consistency in bacterial inoculum density. A 0.5 McFarland standard was prepared by mixing 0.05 mL of 1.175% (w/v) barium chloride dihydrate (BaCl₂·2H₂O) with 9.95 mL of 1% (v/v) sulfuric acid (H₂SO₄). The standard was stored in a dark bottle and vortexed before use. Optical density (OD) measurements at 600 nm were taken to validate bacterial suspensions, ensuring an OD range of 0.08–0.13, corresponding to approximately 1.5 × 10⁸ CFU/mL. Regular calibration checks were performed using a spectrophotometer to maintain accuracy in bacterial concentration.

As a positive control of bacterial growth, inoculated medium was used. The exposed bacteria (*Escherichia coli*, *Staphylococcus aureus*, and *Pseudomonas aeruginosa*) were incubated at 35 °C for 24, 48, and 72 h. Afterward, absorbance was measured in a microplate reader (Thermo Scientific) at 600 nm. Cell culture concentration was determined using Eq. 1.

1 $${\% cell proliferation}= \frac{\text{Sample optical density }}{\text{Positive control optical density}} \times 100 \%$$

### Bioreactor assay

For this assessment, GEL scaffolds were selected to compare bacterial growth within a bioreactor setup. Bacterial cultures were grown in 250-mL Erlenmeyer flasks containing 100 mL of the respective media. The flasks were placed on an orbital shaker set to 150 rpm at 37 °C to ensure proper aeration and homogeneous distribution of nutrients. The dynamic conditions facilitated scaffold interaction with the bacterial cultures, mimicking industrial bioreactor environments. Accordingly, 200 µL of *Pseudomonas aeruginosa, Escherichia coli*, and *Staphylococcus aureus* (each at 0.5 McFarland) were introduced into 50 mL of nutrient broth containing electrospun nanofibers of GEL (500 mg), affixed at the base of 250-mL flasks. Subsequently, these flasks were incubated at 35 °C with consistent agitation (60 rpm) for 24 h. The dry weight was measured using inoculated broth devoid of fibers as a control; this facilitated the calculation of the proliferation percentage. This experimental setup was replicated in triplicate to ensure accuracy and consistency in the results.

## Results

### Scanning electron microscopy (SEM)

**Fig. 1:**
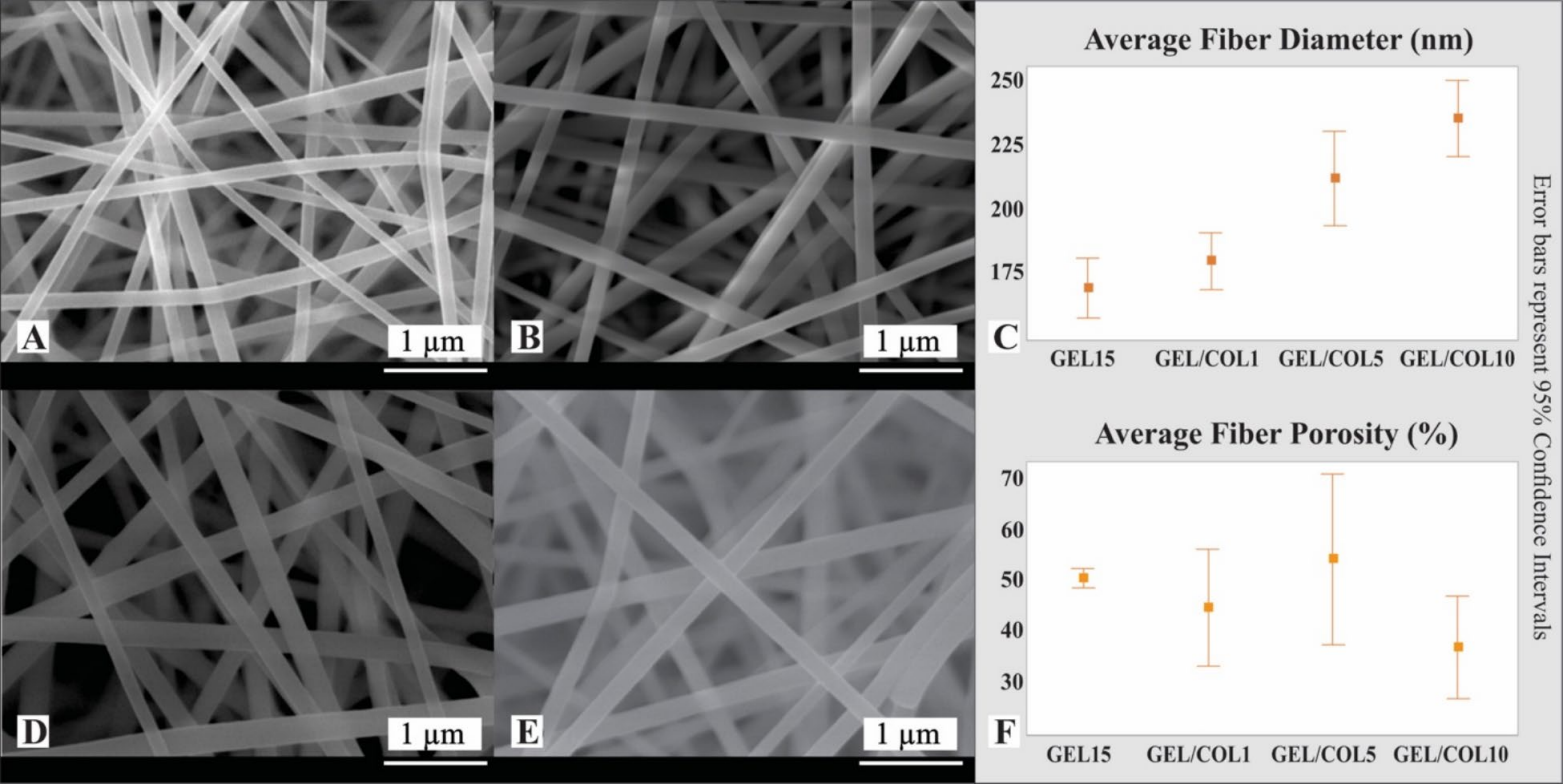
SEM images of electrospun gelatin/collagen scaffolds, showing fiber morphology and uniform distribution. These structures provide a conducive environment for bacterial adhesion and proliferation**.** SEM micrographs of GEL/COL and the average diameter of fibers and the percentage of porosity. **A** GEL at 25 000 ×. **B** GEL/COL1 at 25, 000 ×. **C** Percentage and standard deviation of the diameter of the samples. **D** GEL/COL5 at 25, 000 ×. **E** GEL/COL10 at 25, 000 ×. **F** Percentage and standard deviation of porosity of samples. Error bars represent 95% confidence intervals

SEM micrographs of electrospun samples GEL, GEL/COL1, GEL/COL5, and GEL/COL10 exhibit a smooth surface, indicating the formation of defect-free fibers without cross-linking. The average diameter of the nanofibers ranged from 169 ± 30 nm to 235 ± 40 nm (Fig. 1).

The average porosity percentage varied between 50.04 ± 1.52% and 53.52 ± 13.39%, with the GEL/COL5 sample exhibiting the highest porosity percentage (Table Table 3 Relative LE: Please check if the changes made to Table 3 are appropriate.increase bacterial proliferation percentage in a bioreactor with GEL nanofibers at 35 °C and 24 hStrainProliferation (%)Standard deviation(%)E. coli↑ 400.48S. aureus↑1500.03P. aeruginosa↑ 350.08^1^). Figure 1C showcases the samples’ average diameter and standard deviation, revealing an increase in diameter corresponding to the rise in COL concentration. This trend is also mirrored in the porosity observed in Fig. 1F.

**Table 1:** Average electrospun fiber diameter and average porosity percentage of GEL/COL fibers

GEL/COL fibers	Electrospun fiber diameter	Average porosity percentage
GEL (15% gelatin)	151 ± 31 nm	50.04 ± 1.52%
GEL/COL1	166 ± 28 nm	44.11 ± 9.28%
GEL/COL5	191 ± 40 nm	53.52 ± 13.39%
GEL/COL10	213 ± 36 nm	36.38 ± 8.14%

### Thermogravimetric analysis (TGA) and differential scanning calorimetry (DSC)

**Fig. 2:**
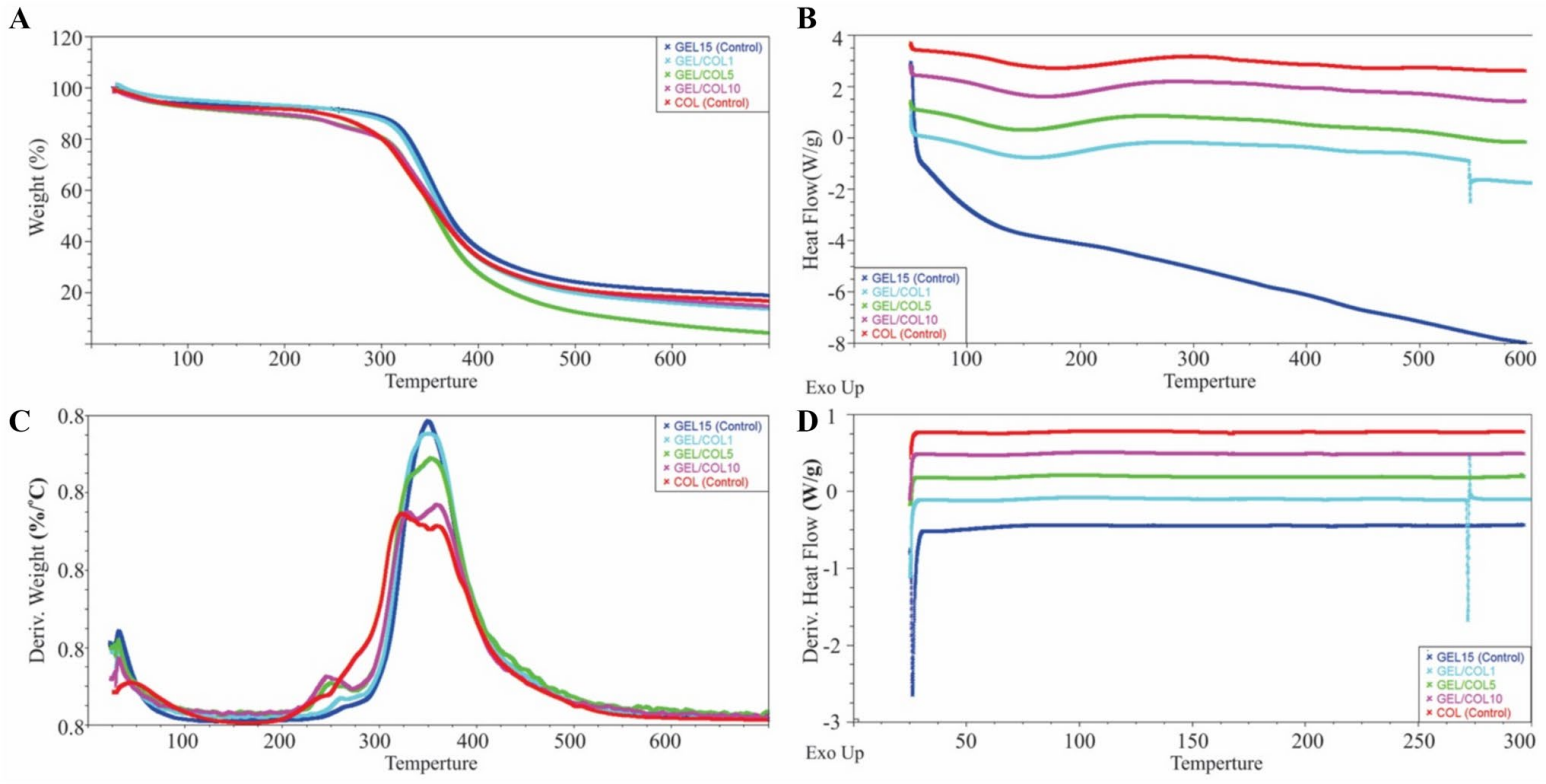
Thermogravimetric analysis regarding the weight percentage of the different GEL and COL membranes. **A** Weight/temperature curve showing the thermal stability of the membranes. **B** The first derivative of the weight/temperature curve indicating degradation stages. **C**, **D** Differential scanning calorimetry analysis of GEL and COL samples, revealing thermal transitions and crystallinity differences

The TGA presented in Fig. 2 displays the degradation curves of GEL/COL1, GEL/COL5, GEL/COL10 samples, and pulverized COL (control), demonstrating thermal degradation attributed to the formation of gaseous products during the reaction.

The GEL/COL10 sample exhibited a notably faster degradation rate compared to the COL control. It experienced a 5% weight loss at 98.39 °C, whereas with less COL, the sample degraded at a similar rate as the control, losing 5% of its weight but at a notably lower temperature (45.62–50.41 °C). The temperature at which 10% weight loss occurred was also measured: GEL exhibited this at 256.37 °C, while for GEL/COL1, GEL/COL5, GEL/COL10, and COL, the temperatures were 189.90 °C, 155.24 °C, 234.35 °C, and 249.68 °C, respectively. The weight loss of 5 to 10% was due to solvent evaporation, which resulted from the boiling point of the acetic acid solvent (118 °C) and the moisture content in the sample.

Moreover, the degradation temperature for 50% weight loss was measured, yielding the following results: GEL at 368.03 °C, GEL/COL1 at 361.07 °C, GEL/COL5 at 355.84 °C, GEL/COL10 at 366.70 °C, and COL at 362.42 °C. At 700 °C, most samples retained a small percentage of their original weight, ranging from 3.83 to 16.90%, while GEL preserved 17.6% of its total weight.

As per the DSC analysis depicted in Fig. 2C and D, evaporation is observed within the range of 73 to 88 °C, likely due to moisture absorption by the fibers. Notably, an interruption is visible in the GEL/COL1 sample, attributed to a system error. In contrast, the GEL sample displays distinct behavior compared to the COL-containing samples, manifesting slight dehydration at 36 °C and transitions at 125 °C, 280 °C, 212 °C, and 259 °C.

### Fourier transform infrared spectroscopy (FTIR)

**Fig. 3:**
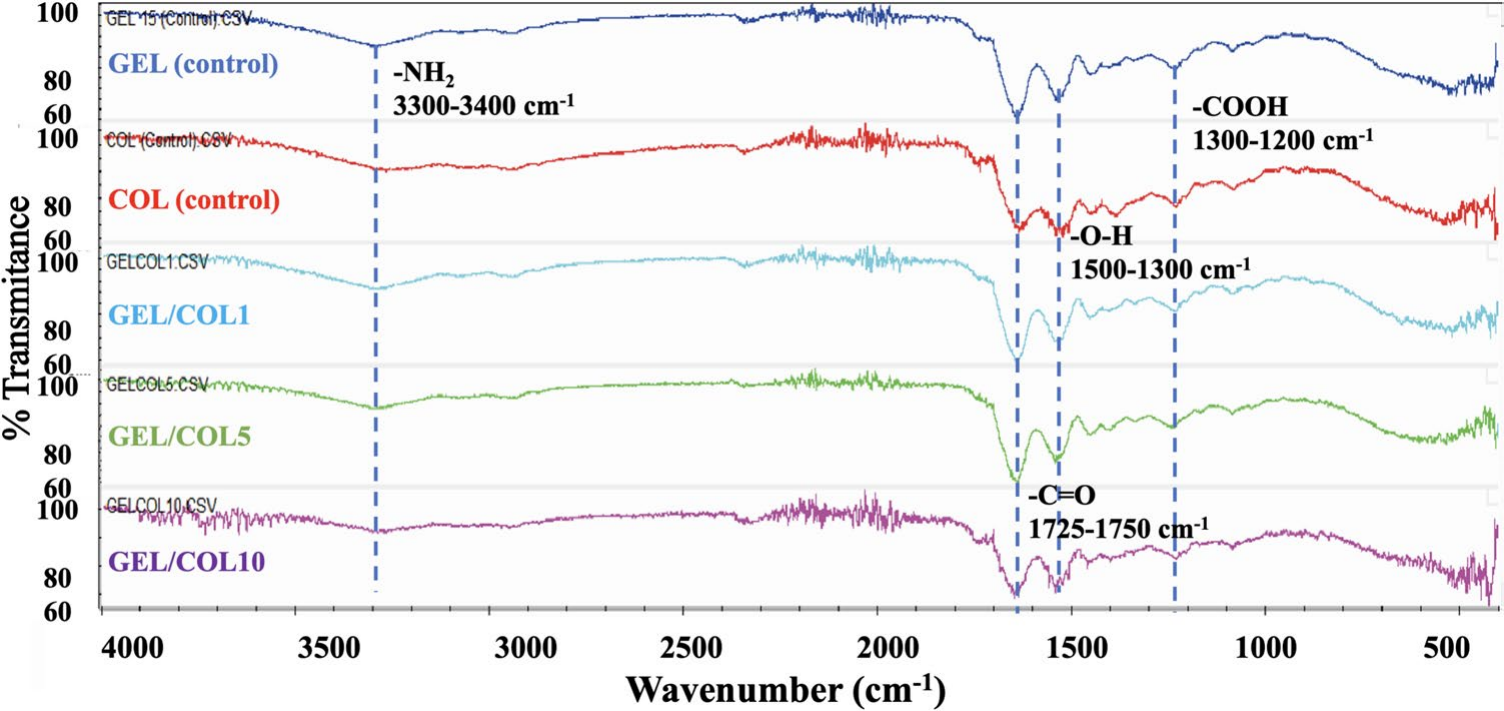
FTIR spectra of the GEL/COL scaffolds with varying gelatin concentrations, illustrating key functional groups associated with protein-based materials and their stability

The FTIR spectra of the samples do not show significant differences. Peaks at 3302 cm^−1^ and around 1300 cm^−1^ correspond to the O–H stretches of the alcohol functional group. Notably, peaks at 1742 cm^−1^ and 1450 cm^−1^ indicate C = O stretches of carbonyl groups, while the peak at 1235 cm^−1^ represents the bending of carboxylic acids. Furthermore, the presence of the functional amine group (-NH_2_) is apparent from N–H stretches observed at 3300–3400 cm^−1^. This group is prevalent in the amino acid chains of COL and GEL, particularly in the basic amino acid Lysine, a key component responsible for the fibrous structure of COL. Additionally, the presence of C-O bonds in secondary alcohols is indicated by a peak at 1100 cm^−1^ (Table 2, Fig. 3).

**Table 2:** Functional groups that can be visualized in FTIR

Functional group	Wavelength (cm^−1^)
O–H (alcohol)	3650–2300, 1500–1300
C = O (carboxylic acid)	1725–1750
-NH_2_ (bending)	3300–3400
-COOH	1300–1200
C-O (a secondary alcohol)	≈ 1

### Cellular proliferation in bacteria

**Fig. 4:**
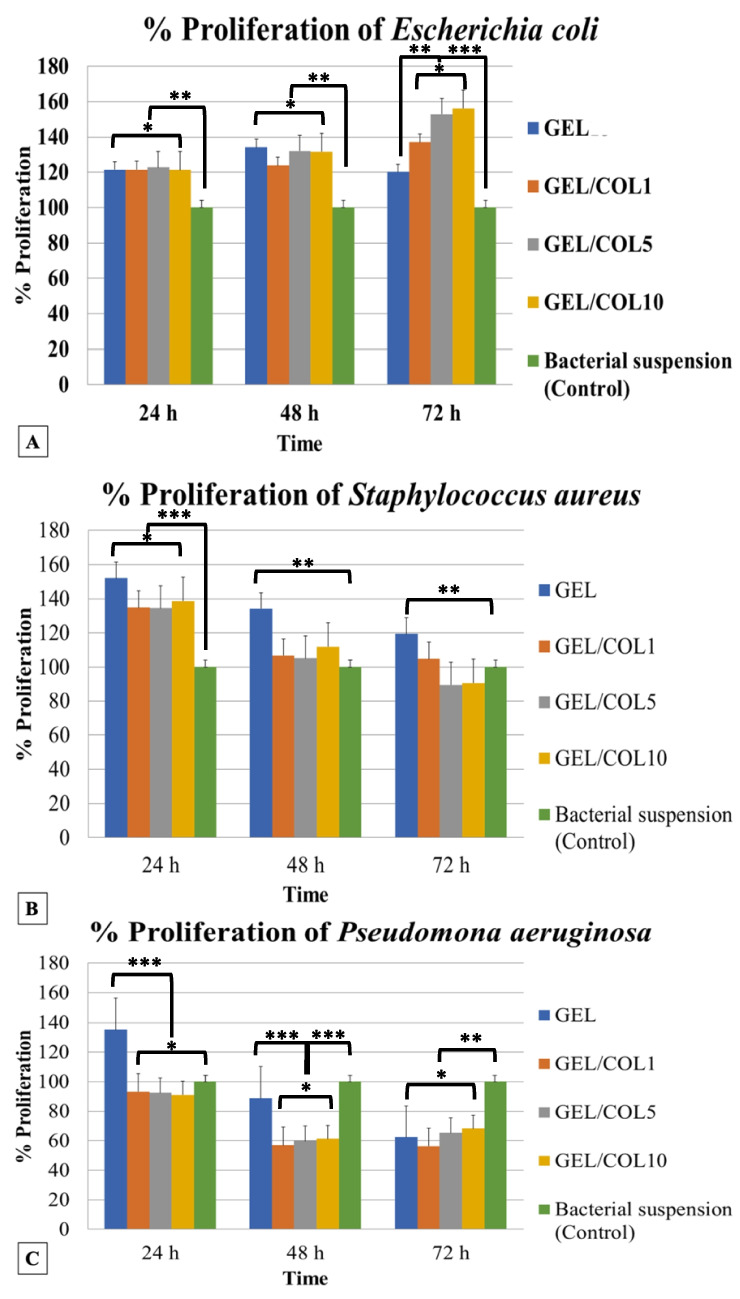
Bacterial proliferation on different scaffold compositions, highlighting the enhanced growth of *Staphylococcus aureus* and selective suppression of *Pseudomonas aeruginosa* under specific conditions. **A** Percentage of proliferation of *Escherichia coli.* **B** Percentage of proliferation of *Staphylococcus aureus.* **C** Percentage of proliferation of *Pseudomonas aeruginosa.* **** value statistically significant when *p* < 0.0001; not statistically significant when *p* > 0.05. *p < 0.05; **p < 0.01; ***p < 0.001; ****p < 0.0001

The absorbance measurements shown in Fig. 4 were taken at 24, 48, and 72 h. They demonstrate that *E. coli* growth was observed in all samples, with at least 20% more growth than the positive control. The latter is particularly evident at 72 h since the percentage of proliferation increases with the increasing amount of COL with a proliferation rate of approx. 55%, compared to the bacterial suspension, is significantly different (ANOVA *p* < 0.05). This suggests the potential suitability of these membranes for various biotechnological or industrial applications (Fig. 4A).

Concerning *S. aureus*, the results after 24 h indicate a significant growth of at least 20% in all nanofiber samples compared to the control (Fig. 4B). After 48 h, the GEL sample stands out as the only membrane showing a substantial 50% increase in proliferation compared to the control. At 72 h, the GEL and GEL/COL1 membranes demonstrated heightened bacterial proliferation compared to the control. However, the effectiveness of the GEL nanofibers diminishes with time, from a 50% proliferation ratio (24 h) to 20% (72 h) compared to the bacterial suspension. Conversely, the GEL/COL5 and GEL/COL10 membranes exhibit less proliferation than the control, possibly due to the elevated collagen concentration.

This decline in effectiveness may be attributed to factors such as nutrient depletion or changes in the microenvironment over time (Galo et al. 2021).

For *P. aeruginosa*, Fig. 4C illustrates the suppression of bacterial growth evident in the GEL/COL1, GEL/COL5, and GEL/COL10 membranes, registering 3% lower proliferation than the control at 24 h. Over 48 and 72 h, all samples displayed reduced bacterial proliferation compared to the positive control. Nevertheless, GEL nanofibers exhibit the highest proliferation rate (35%) among all the samples compared with the bacterial suspension. Additionally, there is a decrease in proliferation as time increases in the GEL samples, as observed with *Staphylococcus aureus bacteria.* This suggests that the fibrous material restricts bacterial proliferation, hinting at potential applications in tissue engineering. This unexpected result suggests that the GEL nanofibers may provide a conducive environment for *P. aeruginosa* growth (LaBauve and Wargo 2012; Jafari et al. 2020).

Interestingly, a decrease in proliferation is observed over time in the GEL samples, similar to the trend observed with *Staphylococcus aureus* bacteria. This temporal decline in bacterial proliferation may be attributed to various factors, including nutrient depletion, microenvironment changes, or nanofiber materials’ inherent properties (Shineh et al. 2023).

### Bioreactor assay

The investigation showed that incorporating GEL nanofibers at the base of a bioreactor-style prototype resulted in enhanced growth for all strains compared to the control of inoculated medium without nanofibers. According to Table 3, *S. aureus* exhibited the highest proliferation rate at 128% (as observed in the cellular proliferation assay, vide supra) followed by *E. coli* at 40%, and finally, *P. aeruginosa* at 34%, higher than the growth observed in the medium inoculated without fibers (control).

**Table 3:** Relative increase bacterial proliferation percentage in a bioreactor with GEL nanofibers at 35 °C and 24 h

Strain	Proliferation (%)	Standard deviation (%)
*E. coli*	↑ 40	0.48
*S. aureus*	↑150	0.03
*P. aeruginosa*	↑ 35	0.08

The provided proliferation data for *Escherichia coli, Staphylococcus aureus*, and *Pseudomonas aeruginosa*, along with comparisons to literature, offers valuable insights into the impact of GEL 15 nanofibers on bacterial growth. *Escherichia coli*, a commonly studied bacterium, displayed a proliferation rate of 40.05%, aligning well with its expected growth characteristics. Typically, *E. coli* exhibits a rapid generation time of around 17 min under optimal conditions, resulting in exponential growth over time. This suggests that the presence of GEL 15 nanofibers may provide a conducive environment for *E. coli* proliferation, albeit at a moderate rate compared to other strains (Tuttle et al. 2021).

In contrast, *Staphylococcus aureus* demonstrated a significantly higher proliferation rate of 128.34%, indicating robust growth in the presence of GEL 15 nanofibers. Literature suggests that *S. aureus* has a longer generation time than *E. coli*, usually ranging from 27 to 30 min. However, the observed proliferation rate surpasses expectations, suggesting that the nanofibers may offer specific benefits that enhance the growth of *S. aureus*. This finding is particularly noteworthy given the clinical relevance of *S. aureus*, a pathogenic bacterium implicated in various infections (Zaharia et al. 2013).

*Pseudomonas aeruginosa*, known for its resilience and versatility, exhibited a proliferation rate of 34.53%, consistent with its longer generation time than *E. coli* and *S. aureus*. Literature suggests that *P. aeruginosa* typically has a generation time of around 75 min. While its proliferation rate was lower compared to the other strains, it still indicates growth in the presence of GEL 15 nanofibers, highlighting the potential utility of these nanofibers in supporting the growth of diverse bacterial species (Qin et al. 2022).

## Discussions

Using extra-pure gelatin and type I fish collagen in electrospun nanofibers offers significant benefits for biotechnological applications, especially in promoting bacterial growth. Both materials are natural biopolymers with excellent biocompatibility, providing a non-toxic environment that supports cellular adhesion and proliferation (El-Seedi et al. 2023).

Gelatin and collagen provide an ideal microenvironment for bacterial growth due to their unique biochemical composition and physicochemical properties. As natural biopolymers, they are rich in peptides and amino acids such as glycine, proline, and hydroxyproline, which serve as essential nitrogen sources for bacterial metabolism (Irastorza et al. 2021). Many bacteria, including *Staphylococcus aureus*, possess specialized collagen-binding proteins such as microbial surface components recognizing adhesive matrix molecules (MSCRAMMs), which enhance their ability to attach to collagen-based scaffolds. This inherent biological affinity not only promotes adhesion but also facilitates robust biofilm formation, a key factor in bacterial proliferation within bioreactors (Kang et al. 2013). Another crucial advantage of gelatin and collagen is their hydrophilicity and high-water retention capacity. These proteins create a moist microenvironment that supports bacterial viability by facilitating nutrient diffusion and oxygen transport (Olteanu et al. 2024). Hydration also plays a significant role in maintaining a dynamic and conducive surface for bacterial proliferation, particularly for biofilm-forming species like *Pseudomonas aeruginosa*. The enhanced diffusion properties of these biopolymers ensure that metabolic by-products are efficiently exchanged, preventing localized depletion of essential growth factors (Vilas Boas et al. 2024).

The electrospun fibers’ resultant fibrous, porous structure mimics the extracellular matrix (ECM) in biological tissues, facilitating bacterial attachment and enhancing nutrient exchange due to the high surface area. This is vital for efficient bacterial growth and essential in processes like fermentation and biocatalysis (Law et al. 2017). Additionally, the combination of gelatin and collagen improves the mechanical stability of the nanofibers, ensuring durability during bacterial cultivation. This blend provides a resilient scaffold for various biotechnological applications (Gao et al. 2016; Sabarees et al. 2024).

The combination of gelatin and collagen in scaffolds allows for better control over degradation rates. Gelatin degrades faster, while collagen provides longer structural stability. This blend can be tuned to support sustained bacterial growth for specific biotechnological processes (Lukin et al. 2022). Their amino acid compositions also enable easy functionalization with bioactive molecules, further optimizing bacterial activity (León-López et al. 2019). Electrospun nanofibers made from extra-pure gelatin and type I fish collagen create effective scaffolds for bacterial growth due to their biocompatibility, enhanced mechanical properties, and controlled degradation (El-Seedi et al. 2023). Furthermore, nanofibers offer a higher surface-area-to-volume ratio, promoting efficient bacterial attachment and nutrient exchange, which improves metabolic activity compared to flat films (Haider et al. 2018).

Nanofibers offer significant advantages due to their high porosity, which enhances the permeability of nutrients, gases, and waste products, promoting bacterial growth. Unlike films, with lower porosity and hinder nutrient diffusion, nanofibers better mimic the natural extracellular matrix (ECM), creating a more supportive environment for bacteria (Jiang et al. 2010). Additionally, nanofibers are mechanically flexible, adapting well to changes during bacterial growth, while films tend to be stiffer (Beachley and Wen 2010) Their high surface area also allows for improved functionalization with bioactive molecules or antimicrobial agents, increasing their versatility for biotechnological applications. (Villarreal-Gómez et al. 2024; Villarreal-Gómez et al. 2024).

On the other hand, gelatin can be used for large-scale industrial bioreactors, and more robust and stable materials like synthetic polymers (e.g., polyethylene glycol), alginate, or other hydrogels are often preferred over gelatin due to its relatively fast degradation rate and variable mechanical properties. Gelatin’s use is generally more prevalent in research, biomedical applications, and niche biotechnological processes rather than in large-scale industrial production (Łabowska et al. 2021).

Determining the porosity of electrospun scaffolds involves analyzing SEM images to measure fiber diameter and pore size. For the GEL/COL10 scaffold, larger fibers lead to reduced inter-fiber space and lower porosity. In comparison, the GEL scaffold has smaller fibers that create more inter-fiber space and higher porosity. Variations in polymer concentration or solvent composition may also affect porosity (Lopez Marquez et al. 2022). In addition to SEM analysis, methods like BET analysis and capillary flow porosimetry can validate porosity measurements. BET assesses specific surface area and pore volume, while capillary flow measures pore size distribution. Combining these techniques provides a more comprehensive assessment for scaffold design and optimization (Peinador et al. 2023).

Using GEL in electrospinning studies has helped identify factors that influence nanofiber morphology (Gao et al. 2016). Adjusting the concentration of the electrospun solution can change nanofiber diameter, while the choice of solvent affects size consistency due to viscosity variations (Jalaja et al. 2016). Solvents like acetic acid maintain stable viscosity, resulting in consistent fiber diameters, while ethanol and hexafluoro- 2-propanol (HFIP) are also commonly used (Aoki et al. 2015). GEL can produce fibers ranging from 2–3 µm to 40–50 nm at 8 to 30% w/v concentrations (Detta et al. 2010; Zha et al. 2012; Angarano et al. 2013; Laha et al. 2016; Safdari et al. 2016; Ghassemi and Slaughter 2018). Even at higher concentrations, the fibers produced show an average diameter of 169 ± 30 nm with a smooth surface and no defects such as bead formation.

Collagen (COL) is not commonly used in electrospinning for nanofiber development. However, some studies have shown promising results with low COL concentrations in fluorinated solvents. For instance, tilapia collagen dissolved in HFIP produced fibers averaging 310 ± 117 nm (Timnak et al. 2011). Additionally, COL and chondroitin sulfate in HFIP resulted in fibers ranging from 50 to 350 nm. Other research suggests using milder solvents like acetic acid with COL and hyaluronic acid, yielding sub- 300 nm fibers (Fischer et al. 2012). Type A collagen from the equine Achilles tendon generated fibers roughly 100 µm in diameter (Zulkifli et al. 2023), but pure COL samples were difficult to achieve due to low solution viscosity.

Hofman et al. (2012) examined electrospun COL and gelatin (GEL) using acetic acid for GEL and HFIP for COL. The resulting fiber diameters ranged from 100 to 200 nm, aligning with our measurements (179 ± 30 nm for GEL/COL1, 211 ± 49 nm for GEL/COL5, and 235 ± 40 nm for GEL/COL10), demonstrating effective dissolution and avoiding aggressive solvents (Hofman et al. 2012).

Thermogravimetric analysis (TGA) and differential scanning calorimetry (DSC) are key techniques for assessing the thermal properties of materials, including the presence of collagen in gelatin fibers (Samouillan et al. 2011). The sample’s weight is monitored during heating, revealing distinct thermal degradation profiles for collagen and gelatin due to their molecular differences. Collagen undergoes degradation in stages—water loss, denaturation, and decomposition—while gelatin, derived from collagen, shows altered degradation behavior (Bozec and Odlyha 2011). By comparing TGA curves of gelatin fibers with and without collagen, variations in thermal degradation, such as additional weight loss events or shifts in degradation temperature, can indicate collagen presence (Renkler et al. 2023).

The thermogravimetric analysis demonstrated that the GEL/COL1, GEL/COL5, and GEL/COL10 samples experienced high-temperature degradation (155 to 234 °C), whereas the control temperatures were 256 °C for GEL and 249 °C for COL. As suggested by prior studies (Laha et al. 2016; Zhou et al. 2016), this property holds promise for potential biomedical applications.

According to León-Mancilla et al. (2016), COL samples undergo three phases of weight loss: first due to water loss, second attributed to COL combustion at 311.63 °C, and third from 450 to 700 °C, where approximately 25% of the combustion residue remains (León-Mancilla et al. 2016). T. Zhou et al. (2016) observed COL combustion occurring between 190 and 600 °C; in our study, the control COL exhibited a combustion temperature of 324 °C based on the weight derivative concerning temperature (Zhou et al. 2016). According to Mukherjee and Rosolen (2013), measurements suggest that the combustion temperature of GEL initiates below 200 °C (Mukherjee and Rosolen 2013). In contrast, as per Laha et al. (2016), degradation levels peak between 291 and 500 °C, leaving 24% of the weight as residual waste. However, our GEL demonstrated significantly higher stability at elevated temperatures, exhibiting a peak around 350 °C and retaining a residual percentage of 22% at 500 °C (Laha et al. 2016).

Similarly, DSC can reveal the thermal transitions of gelatin fibers containing collagen, such as denaturation and melting, affecting the DSC thermogram’s onset temperature and peak shape (Leyva-Porras et al. 2019). TGA and DSC together provide complementary insights into the thermal properties of gelatin fibers, confirming collagen presence and aiding in the characterization of gelatin-based materials for biomedical and industrial applications (Deng et al. 2018); Patarroyo et al. 2020; McLeod et al. 2024).

For León-Mancilla et al. (2016), the COL sample exhibits three peaks. The first endothermic peak ranged between 85 and 90 °C (León-Mancilla et al. 2016). However, in the Foltran et al. (2008) study, this peak was noted at 76 °C and 112 °C for completely dried samples (Foltran et al. 2008). In our samples, the endothermic degradation due to water evaporation occurred between 76 and 88 °C, with a rise in temperature corresponding to increased COL concentration. The second transition, another endothermic peak linked to protein denaturation, typically occurs between 275 and 325 °C. While some studies suggest a narrower range for this second peak (Bozec and Odlyha 2011), indicating a transition between 305 ± 10 °C and 345 ± 10 °C, it was not observable in Fig. 2C and D. The final transition was an exothermic peak due to COL combustion occurring between 350 and 425 °C. This peak was not visible in our analysis, which concluded at 300 °C; however, these temperatures were captured in the TGA analysis.

Acetic acid CH_3_-COOH typically shows a distinct peak in FT-IR at 1706 cm^−1^ for the C = O bond (Safdari et al. 2016). However, our FTIR analysis did not detect this peak, indicating the absence of acetic acid in the samples.

León-Mancilla et al. (2016) identified a peak at 3285 cm^−1^ in COL, corresponding to the amide A (N–H bond) (León-Mancilla et al. 2016). Muyonga et al. (2004) noted that GEL samples had a broader amide peak and an additional peak at 2930 cm^−1^, indicating stable dimeric associations of carboxylic acids. The glycosylation of COL samples may have occurred during electrospinning (Muyonga et al. 2004).

In the COL sample, peaks were noted at 3500–3000 cm^−1^ and 1100–1000 cm^−1^ due to carbohydrate glycosylation. Zha et al. (2012) and Sisson et al. (2009) reported amide peaks between 3290 and 3306 cm^−1^ and 3300 cm^−1^, respectively, while our analysis found a prominent peak at 3302 cm^−1^ that slightly shifted with increased COL concentration. A peak at 1083 cm^−1^, linked to carbohydrates in COL, was absent in GEL samples (Sisson et al. 2009; Zha et al. 2012).

Observed peak shifts can be linked to variations in molecular interactions within the samples. Increasing collagen concentration may alter the local chemical environment and interactions with other components, like gelatin. These changes can affect the vibrational frequencies of functional groups, leading to peak shifts in FTIR spectra (Freudenberg et al. 2007). For instance, shifts may result from changes in hydrogen bonding, conformational states, or the chemical environments of specific groups due to differences in intermolecular association, cross-linking density, or interactions from varying collagen concentrations (Sionkowska et al. 2009).

According to various studies (Foltran et al. 2008; Zha et al. 2012; Lu et al. 2015; Erencia et al. 2015; Jalaja et al. 2016; Laha et al. 2016; León-Mancilla et al. 2016; Safdari et al. 2016; Ma et al. 2016), amide II typically exhibits a peak from 1560 to 1535 cm^−1^ due to interactions involving nitrogen and hydrogen, as well as carbon and hydrogen. Our study detected this peak at approximately 1536 cm^−1^ for both the GEL and COL samples before and after the electrospinning process.

Studies confirm that the amide III band associated with the β-sheet structure of collagen is between 1235 and 1240 cm^−1^, with this study identifying it at 1236 cm^−1^, indicating hydrogen bonding for the β-structure (Foltran et al. 2008; Zha et al. 2012; Lu et al. 2015; Erencia et al. 2015; Jalaja et al. 2016; Laha et al. 2016; León-Mancilla et al. 2016; Safdari et al. 2016; Ma et al. 2016). The minimal differences between the samples and controls of GEL and COL suggest no significant structural changes occurred during the electrospinning process, preserving their structure.

In conclusion, the FT-IR analysis did not reveal the presence of any residual solvent. The observed peaks were assigned to the amide A peak (N–H) in both the GEL and COL samples, which were found between 3500 and 3000 cm⁻^1^. However, we cannot rule out the possibility of a glycosylation process occurring during electrospinning. Additionally, the amide II peaks were observed in both GEL and COL samples within the range of 1560 to 1535 cm⁻^1^, with no changes detected during the electrospinning process. The presence of the amide group was confirmed in all the samples by observing the v(C = O) band.

Regarding bacterial proliferation, our results indicate that collagen in membranes enhances *E. coli* growth, showcasing their potential for biotechnological applications. This enhanced growth could be due to collagen’s biocompatibility, providing a favorable environment for bacterial adhesion and possibly serving as a nutrient source (Zhang et al. 2023).

The significant difference in proliferation rates between collagen-containing samples and bacterial suspension (ANOVA, *p* < 0.05) emphasizes the role of collagen membranes in promoting bacterial growth. These findings could impact tissue engineering, wound healing, and bioremediation. Further research is essential to understand the mechanisms and optimize collagen formulations for specific applications, advancing our knowledge of bacterial and biomaterial interactions (Dutta et al. 2021).

The findings on *S. aureus* highlight the complex relationship between biomaterial composition and bacterial behavior. While GEL may initially encourage bacterial growth, its effectiveness decreases over time. Conversely, GEL/COL1 membranes with collagen support longer-term bacterial proliferation, enhancing our understanding of biomaterial impacts on bacterial dynamics and guiding future optimization (Takallu et al. 2024).

Finally, for *P. aeruginosa*, GEL nanofibers can either restrict or promote growth, suggesting potential applications in tissue engineering for regulating bacterial colonization to aid tissue regeneration or prevent infection. Further research is necessary to understand these effects on nanofiber membranes and their biomedical applications (Gul et al. 2021).

Our group has focused on studying bacterial mat formations for biotechnological applications (Velasco-Barraza et al. 2018; Pompa-Monroy et al. 2020, 2022), which sets us apart from most studies that emphasize antibacterial activity (Augustine et al. 2016; Álvarez-Suárez et al. 2020; Gul et al. 2021; Chiloeches et al. 2022). To effectively compare our results with existing literature, we must consider factors such as bacterial growth rates, material composition, and the specific applications of the formulations (Bonnet et al. 2020) (see Table 4). Notably, there is limited research on the use of electrospun fibers to enhance bacterial growth, making our work pioneering in this field.

**Table 4:** Comparative studies from our group of electrospun fibers proposed for biotechnological purposes

Study	Effective formulation	Bacterial proliferation	Electrospun fiber diameter	Application	Reference
GEL/COL Nanofibers	GEL (15% gelatin)	150% proliferation of *S. aureus* in 24 h	151 ± 31 nm	Bacterial cultivation for biotechnology	Present study
	GEL/COL1	138% proliferation of *E. coli* in 72 h	166 ± 28 nm		
	GEL/COL5	160% proliferation of *E. coli* in 72 h	191 ± 40 nm		
	GEL/COL10	175% proliferation of *E. coli* in 72 h	213 ± 36 nm		
PAA, PAA/CS, and PAA/ALG Nanofibers	PAA/ALG (90:10)	127% proliferation of *Streptomyces spp*., in 24 h	339 ± 61 nm	Biotechnological applications	(Velasco-Barraza et al. 2018)
PCL/Curcumin Nanofibers	PCL/CUR3 (5% Curcumin) (10% PCL)	137% proliferation of *E. coli* in 12 and 24 h	441 ± 154 nm	Biotechnological applications	(Pompa-Monroy et al. 2020)
PCL/Cs and PCL/Ns nanofibers	PCL/Lac and PCL/Gly fibers (13% PCL) (0.16% Lac and Gly)	~ 153% and ~ 142% proliferation of *S. aureus* in 48 h	313 to 766 nm	Water bioremediation	(Pompa-Monroy et al. 2022)

*ALG* alginate, *COL* collagen, *CS* chitosan, *Cs* carbon source, *GEL* gelatin, *Gly* glycine, *Lac* lactose, *PAA* poly (acrylic acid), *PCL* polycaprolactone, *Ns* nitrogen source.

The GEL/COL nanofibers in this study showed a 128% proliferation of *Staphylococcus aureus* over 72 h, indicating their potential for bacterial cultivation in biotechnological applications. In contrast, PAA/ALG nanofibers (90:10) displayed a 127% increase in *Streptomyces* spp. growth in just 24 h, showcasing their efficiency in applications requiring rapid microbial growth (Velasco-Barraza et al. 2018).

The PCL/curcumin nanofibers showed a proliferation rate of 137% for *E. coli* in 12 to 24 h, indicating their potential for rapid bacterial cultivation. In contrast, PCL/Lac and PCL/Gly fibers achieved approximately 153% proliferation of *S. aureus* over 48 h, presenting another strong option for bacterial growth enhancement. These findings highlight the versatility of nanofibrous materials in microbial applications (Pompa-Monroy et al. 2020).

GEL/COL nanofibers have a diameter of 300–500 nm, which is favorable for bacterial interaction, similar to the PAA/ALG fibers at 339 ± 61 nm. Both types support bacterial adhesion and growth. In contrast, PCL/curcumin fibers are larger at 441 ± 154 nm, while PCL/Lac and PCL/Gly fibers range from 313 to 766 nm. These diameter differences may affect surface properties and bacterial dynamics (Pompa-Monroy et al. 2022).

This current research focuses on bacterial cultivation for biotechnology, highlighting the effectiveness of PAA/ALG fibers for various biotechnological applications (Velasco-Barraza et al. 2018). PCL/curcumin fibers are also suitable for microbial studies (Pompa-Monroy et al. 2020), while PCL/Lac and PCL/Gly fibers are specifically designed for water bioremediation, demonstrating the diverse potential of different nanofiber formulations (Pompa-Monroy et al. 2022).

The analysis shows that GEL/COL nanofibers are competitive in promoting bacterial proliferation for biotechnological applications. While some studies report higher growth rates, the GEL/COL nanofibers demonstrate strong potential for effective bacterial cultivation over 72 h, indicating promising opportunities for robust growth in biotechnological contexts.

The bioreactor assay highlights the effectiveness of GEL 15 nanofibers in promoting *Staphylococcus aureus* growth. This finding has implications for biomedical and biotechnological applications that require bacterial proliferation. Further research is needed to explore the underlying mechanisms for optimizing nanofiber platforms (Haider et al. 2018; Larue et al. 2024).

The remarkable enhancement in *Staphylococcus aureus* growth observed in the bioreactor assay (128%) suggests that the gelatin/collagen (GEL/COL) scaffolds provide a highly favorable environment for bacterial proliferation. Several potential mechanisms could explain this effect, including direct interactions between *S. aureus* and gelatin/collagen, as well as the role of these scaffolds as a nutrient source (Wang et al. 2023).

Collagen and gelatin, both structural proteins, influence bacterial adhesion and growth. *S. aureus* has collagen-binding adhesins, like MSCRAMMs, which help it attach to extracellular matrix proteins, enhancing colonization and biofilm formation. Lysostaphin, associated with *S. aureus*, may also facilitate bacterial survival through collagen interactions (Kang et al. 2013). Gelatin, being a hydrolyzed collagen, promotes bacterial adhesion due to its solubility and availability of peptide sequences recognized by *S. aureus* adhesins. This interaction likely leads to high proliferation rates with GEL scaffolds, especially in bioreactor assays with constant agitation (Madani et al. 2017).

Gelatin/collagen scaffolds may enhance *S. aureus* growth by providing a supplementary nutrient source. During incubation, gelatin hydrolysis releases essential peptides and amino acids like glycine and proline, supporting bacterial metabolism and biofilm formation (Madani et al. 2017). Furthermore, the porous structure of electrospun nanofibers facilitates nutrient retention, creating microenvironments with concentrated metabolites. This capability may promote faster cell division and higher biomass accumulation, especially in the dynamic conditions of a bioreactor, compared to static culture assays (Lu et al. 2024).

The increase in *S. aureus* growth indicates that gelatin/collagen nanofibers may be effective substrates for microbial bioreactors in biotechnology. These scaffolds can enhance bacterial proliferation, potentially enabling the production of important bioproducts. Additionally, studying the interactions between bacteria and biomaterials can help optimize scaffold composition for specific microbial cultures (Urso et al. 2024).

Interestingly, suppressing *Pseudomonas aeruginosa* growth in certain nanofiber conditions raises questions about whether it’s a limitation or an exploitable feature. This reduced proliferation may stem from factors such as the nanofiber composition, nutrient availability, and bacterial response mechanisms (LaBauve and Wargo 2012). Gelatin/collagen scaffolds may create a microenvironment that is less favorable for *P. aeruginosa* adherence and biofilm formation, as it lacks the affinity for gelatin-based substrates that *S. aureus* has for collagen. Additionally, if the scaffold mainly provides nutrients favoring *S. aureus*, *P. aeruginosa* may not receive enough resources for optimal growth (Oliveira et al. 2023; Zhou et al. 2023).

From a biotechnological perspective, suppressing *P. aeruginosa* can be beneficial when selective bacterial growth is needed, such as in processes relying on *S. aureus*-derived products. Conversely, if *P. aeruginosa* is required, modifications like surface functionalization or alternative polymer compositions can optimize its growth (Shah et al. 2024). Future research should focus on adjusting scaffold properties—such as porosity, cross-linking density, and bioactive molecule incorporation—to better control microbial interactions in nanofiber-based bioreactors (Serrano-Aroca et al. 2022; Shishparenok et al. 2024).

Using GEL/COL fibers has notable advantages. According to Grover et al. (2012), non-cross-linked nanofibers degrade completely in about 1 h in PBS. Additionally, non-cross-linked gelatin-collagen scaffolds lose 85% of their mass after 14 days of incubation, eliminating the need for a separation process to collect by-products of bacterial metabolism from the bioreactor (Grover et al. 2012).

The discrepancy between the cellular proliferation assay and the bioreactor assay results for *Pseudomonas aeruginosa* may stem from differing experimental conditions and the dynamic nature of bacterial growth. The controlled laboratory conditions of the cellular assay contrast with the more complex environment of the bioreactor, which could have provided growth-promoting factors or facilitated interactions with other microorganisms, resulting in enhanced proliferation (Konopacki et al. 2022).

The variability and limitations of each assay, such as sampling techniques and sensitivity, may have impacted the results. While the cellular proliferation assay offers insights into bacterial growth in controlled conditions, the bioreactor assay provides a broader understanding of bacterial behavior in complex environments. Thus, careful interpretation and validation across multiple assays are crucial for understanding the mechanisms behind *P. aeruginosa* proliferation and its growth dynamics (Adan et al. 2016).

**Fig. 5:**
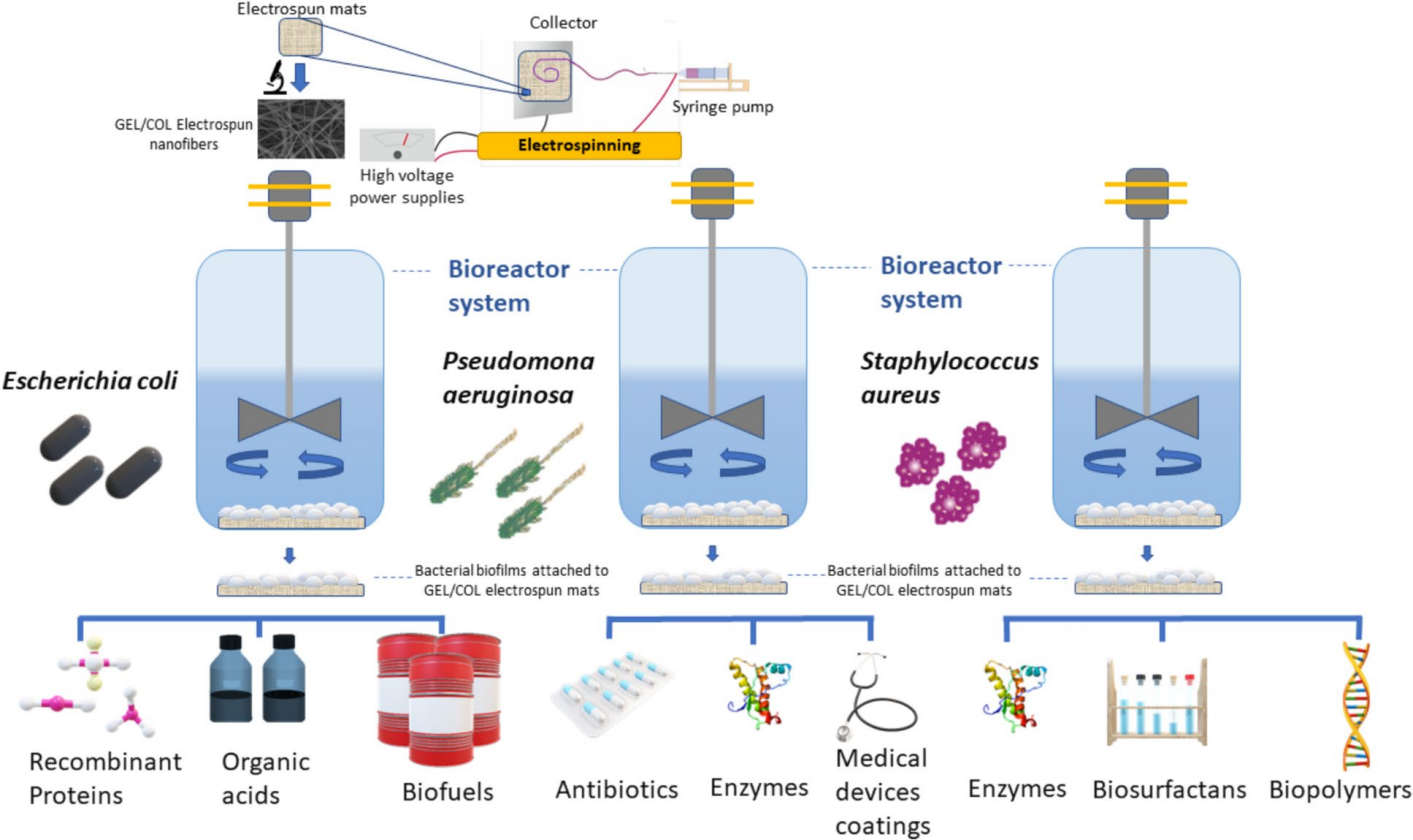
Potential industrial products derived from the bacterial mats grown in GEL/COL electrospun scaffolds

*Escherichia coli*, *Staphylococcus aureus*, and *Pseudomonas aeruginosa* are bacteria with important industrial applications in biotechnology and bioprocessing (Araujo et al. 2022) (Fig. 5). *E. coli* is commonly used for producing recombinant proteins, enzymes, and hormones due to its rapid growth and well-defined genetics (Fakruddin et al. 2013). It’s also utilized in biofuel production, metabolizing sugars from biomass to create alternatives like ethanol and butanol, supporting sustainable energy (Koppolu and Vasigala 2016). Additionally, engineered *E. coli* strains can produce organic acids such as acetic, lactic, and succinic acid, which have various uses in food, pharmaceuticals, and biodegradable plastics (Mazumdar et al. 2010; Dulnik et al. 2016).

Certain strains of *Staphylococcus aureus* produce enzymes like lipases and proteases, which are used in industries such as food processing, detergent manufacturing, and bioremediation (Raveendran et al. 2018). While *S. aureus* is often linked to antibiotic resistance, some strains produce antibiotics like aureomycin, used in agriculture and clinical treatments (Xu et al. 2022). Additionally, *S. aureus* bacterial mats have applications in medical device manufacturing, offering coatings to prevent bacterial colonization on implants (Idrees et al. 2021).

Finally, *Pseudomonas aeruginosa* strains produce various biopolymers like alginate and rhamnolipids. Alginate is valued in food, pharmaceuticals, and wound care for its gelling and emulsifying properties, while rhamnolipids are used in bioremediation, enhanced oil recovery, and personal care products (Soberón‐Chávez et al. 2021). Additionally*, P. aeruginosa* produces biosurfactants utilized in cosmetics, agriculture, and environmental cleanup by emulsifying hydrophobic substances and degrading pollutants (Ambaye et al. 2021). Certain strains also synthesize industrial enzymes like oxidases, dehydrogenases, and lipases, which serve as biocatalysts in the production of pharmaceuticals, fine chemicals, and biodegradable polymers (Timnak et al. 2011). Overall, *Escherichia coli*, *Staphylococcus aureus*, and *Pseudomonas aeruginosa* provide a wealth of valuable applications in biotechnology and bioprocessing.

Future research should focus on optimizing gelatin/collagen scaffolds for long-term bioreactor applications, ensuring their structural stability and sustained bacterial support over extended operational cycles. Studies should investigate advanced cross-linking strategies or composite formulations to enhance mechanical resilience while maintaining biodegradability (Dhand et al. 2016; Zhao et al. 2021). Additionally, optimizing scaffold composition and architecture through nanostructuring or surface modifications could improve bacterial adhesion, nutrient exchange, and metabolic efficiency. Scaling up these bioreactors requires process engineering approaches to integrate gelatin/collagen scaffolds into industrial fermentation systems, assessing their efficiency in large-scale microbial production of biofuels, enzymes, and biopolymers (Samyn et al. 2023). Life cycle assessments (LCAs) should also be conducted to quantify the environmental impact of using gelatin/collagen scaffolds compared to synthetic alternatives, evaluating their carbon footprint, resource consumption, and waste reduction potential. Furthermore, interdisciplinary research combining biomaterials science, microbiology, and process engineering will be essential to fully harness the potential of these biopolymers in sustainable biomanufacturing (Rudolf et al. 2024).

The use of collagen and gelatin (COL/GEL) nanofibers often involves repurposing natural sources from industries like food processing and aquaculture, aligning sustainability principles and offering economic and environmental benefits (Coppola et al. 2020). Collagen and gelatin, sourced from animal by-products such as bones and skin, are prevalent in meat processing and fish farming. By converting these waste materials into COL/GEL nanofibers, researchers support the circular economy, enhancing resource efficiency and reducing environmental impact. This repurpose not only adds value to low-value materials but also creates new revenue streams while minimizing the demand for virgin resources and associated pollution from waste disposal (Rajabimashhadi et al. 2023).

Incorporating this sustainable perspective of COL/GEL nanofibers in bacterial bioreactor discussions emphasizes their eco-friendly potential and encourages interdisciplinary collaborations across biotechnology, food science, and environmental sustainability (Prokisch et al. 2023).

Applications in bioreactors can be improved by incorporating cross-linkers into COL/GEL nanofibers; however, this method has both advantages and disadvantages for bioreactor uses. It enhances stability and mechanical strength, offering better support for bacterial growth and ensuring structural integrity over time. This stability can reduce substrate replacement frequency and improve bacterial cultivation efficiency. Additionally, controlled degradation from cross-linking aids in collecting bacterial by-products and streamlining bioreactor workflows (Furuike et al. 2016; Campiglio et al. 2019).

Cross-linking in COL/GEL nanofibers can present some potential drawbacks. When cross-linking is excessive, it may reduce the biodegradability of the nanofibers, which can hinder their resorption and remodeling by the body. This reduction can negatively impact tissue regeneration in biomedical applications. Moreover, cross-linking can lead to changes in the mechanical properties of the nanofibers, such as increased stiffness, which may compromise the flexibility essential for optimal bacterial adhesion in bioreactors. Therefore, it is crucial to carefully consider the degree of cross-linking in COL/GEL nanofibers to enhance their effectiveness while minimizing these drawbacks (Li et al. 2021). This cross-linking approach remains unexplored, but it could be novel for future research.

While this study did not compare GEL/COL fibers with films for bacterial proliferation, a key benefit of electrospun nanofibers is their high surface area-to-volume ratio, which promotes bacterial adhesion and growth. This feature allows for improved nutrient exchange, fostering an environment conducive to bacterial proliferation. In contrast, films have a limited surface area that restricts nutrient accessibility essential for robust bacterial growth (El-Seedi et al. 2023).

Electrospun nanofibers have a porous structure that enhances the permeability of nutrients, gases, and waste, closely resembling natural extracellular matrices. This encourages better bacterial colonization and growth patterns compared to GEL or COL films, which lack porosity and may restrict bacterial growth due to limited nutrient exchange (Chen et al. 2024). Experimental results highlight the effectiveness of electrospun GEL fibers containing 15% gelatin, which supported significant bacterial proliferation. For example, *Staphylococcus aureus* exhibited over 100% growth within 24 h on these nanofibers, outperforming films, which provide a less dynamic environment. The superior growth rates are linked to the nanofibers’ ability to create an optimal microenvironment for bacterial cultures (Dadras Chomachayi et al. 2018).

Combining GEL and COL in nanofibers allows for tunable degradation rates, promoting sustained bacterial growth in dynamic environments like bioreactors. In contrast, GEL or COL films degrade more slowly and lack the necessary adaptability for applications requiring structural changes. Nanofibers are thus more suitable for continuous bacterial proliferation. Furthermore, electrospun nanofibers demonstrated significant advantages in bioreactor setups, with GEL nanofibers achieving up to 150% proliferation for *Staphylococcus aureus*, outperforming controls and traditional films. While films are durable, they are less effective in dynamic conditions, making nanofibers superior due to their structural and interactive properties.

This study investigated four variations of GEL/COL nanofiber scaffolds for stimulating bacterial growth in bioreactors. The nanofibers, with diameters between 169 and 235 nm and about 50% porosity, demonstrated good chemical stability. Increasing collagen concentration produced thicker fibers without compromising thermal stability. Notably, GEL (15% gelatin) significantly enhanced *Staphylococcus aureus* growth, achieving over 100% proliferation in 24 h, while higher collagen levels in GEL/COL5 and GEL/COL10 reduced bacterial growth. The findings highlight the effectiveness of GEL/COL nanofibers as a substrate for cultivating bacteria, especially in biotechnological applications. Bacterial proliferation assays with *Escherichia coli*, *Staphylococcus aureus*, and *Pseudomonas aeruginosa* confirmed their potential for enhancing bacterial cultures.

## Data Availability

The datasets and materials from this study can be available upon request to interested researchers.
